# Loss of Ventricular Function After Bidirectional Cavopulmonary Connection: Who Is at Risk?

**DOI:** 10.1007/s00246-020-02433-9

**Published:** 2020-08-11

**Authors:** Marie Vincenti, M. Yasir Qureshi, Talha Niaz, Drew K. Seisler, Timothy J. Nelson, Frank Cetta

**Affiliations:** 1 Todd and Karen Wanek Program for Hypoplastic Left Heart Syndrome, Rochester, MN USA; 2 Division of Pediatric Cardiology, Mayo Clinic, 200 First Street S.W, Rochester, MN 55905 USA

**Keywords:** Single ventricle, Bidirectional cavopulmonary connection, Hypoplastic left heart syndrome, Heart failure

## Abstract

Decline of single ventricle systolic function after bidirectional cavopulmonary connection (BDCPC) is thought to be a transient phenomenon. We analyzed ventricular function after BDCPC according to ventricular morphology and correlated this evolution to long-term prognosis. A review from Mayo Clinic databases was performed. Visually estimated ejection fraction (EF) was reported from pre-BDCPC to pre-Fontan procedure. The last cardiovascular update was collected to assess long-term prognosis. A freedom from major cardiac event survival curve and a risk factor analysis were performed. 92 patients were included; 52 had left ventricle (LV) morphology and 40 had right ventricle (RV) morphology (28/40 had hypoplastic left heart syndrome (HLHS)). There were no significant differences in groups regarding BDCPC procedure or immediate post-operative outcome. EF showed a significant and relevant decrease from baseline to discharge in the HLHS group: 59 ± 4% to 49 ± 7% or − 9% (*p* < 0.01) vs. 58 ± 3% to 54 ± 6% or − 4% in the non-HLHS RV group (*p* = 0.04) and 61 ± 4% to 60 ± 4% or − 1% in the LV group (*p* = 0.14). Long-term recovery was the least in the HLHS group: EF prior to Fontan 54 ± 2% vs. 56 ± 6% and 60 ± 4%, respectively (*p* < 0.01). With a median follow-up of 8 years post-BDCPC, six patients had Fontan circulation failure, four died, and three had heart transplantation. EF less than 50% at hospital discharge after BDCPC was strongly correlated to these major cardiac events (HR 3.89; 95% Cl 1.04–14.52). Patients with HLHS are at great risk of ventricular dysfunction after BDCPC. This is not a transient phenomenon and contributes to worse prognosis.

## Introduction

The bidirectional cavopulmonary connection (BDCPC) has been utilized as an intermediate procedure prior to Fontan for patients with functionally single ventricle (SV) hearts since the 1980s [1]. The BDCPC is usually performed when a child is 3 to 6 months of age. While many studies have assessed risk factors within the “interstage period” between the index procedure (Norwood operation for hypoplastic left heart syndrome (HLHS)) and BDCPC [2–5] or after Fontan [6–8]; few studies have focused on the period between BDCPC and Fontan.

The BDCPC is thought to have the least morbidity and mortality and is generally associated with a short cardiopulmonary bypass time [9]. However, the BDCPC changes preload parameters of the SV and could impact ventricular function [10] thereby impacting candidacy for Fontan operation and long-term outcome [11]. Sustaining good ventricular function is one of the main goals of management of patients with a functionally SV heart [12, 13].

This study aimed to evaluate ventricular function before and after BDCPC according to ventricular morphology using visually estimated ejection fraction. Our hypothesis was that, after BDCPC, ventricular dysfunction was associated with right ventricular (RV) morphology. Our second goal was to evaluate survivorship from major cardiac events after BDCPC and to identify the risk factors for poor long-term prognosis. Our hypothesis was that the post-BDCPC ventricular dysfunction was associated with worse long-term prognosis.

## Methods

We conducted an IRB-approved, retrospective study using chart reviews from the Mayo Clinic SV, HLHS, BDCPC, and Fontan databases. All patients consented for the use of medical records. We included all patients with a functionally SV heart who had a BDCPC at Mayo Clinic (Rochester, MN) between 1997 and 2017. All patients included in this study had pre- and post-BDCPC imaging data available to assess the ventricular function. We created two groups according to the SV morphology: Left ventricle (LV) morphology (LV group) and RV morphology (RV group). The RV group was then subdivided into HLHS (HLHS group) and non-HLHS (non-HLHS RV group). Demographic data, surgical management data, laboratory studies, and echocardiography measurements were collected to assess differences between groups. Catheterization measurements were reported pre-BDCPC and pre-Fontan. Volumetric EF obtained during catheterization was calculated using the stroke volume method as follow: EF = (end-diastolic volume (EDV) − end-systolic volume (ESV))/EDV.

Among the echocardiographic data, atrioventricular valve (AV) regurgitation was visually estimated as: 0 = none or trivial, 1 = mild, 2 = moderate and 3 = severe. Aortic arch obstruction was assessed according to mean systolic Doppler gradient in the descending thoracic aorta as: 0 = none (< 5 mmHg), 1 = mild (5–14 mmHg), 2 = moderate (15–24 mmHg), 3 = severe (≥ 25 mmHg).

### Ventricular Function Analysis

We used 2D echocardiographic visually estimated ejection fraction (EF) to assess ventricular function. This is a semi-quantitative measurement of systolic ventricle function by an expert sonographer, comparable to biplane auto-EF [14] and magnetic resonance imaging-EF [15] and usable for both ventricle morphologies. Visually estimated EF was evaluated using several orthogonal planes. It was assessed by sequential evaluation at pre-BDCPC (baseline), hospital discharge, 1 to 3 months post-BDCPC, 6 to 12 months post-BDCPC, and immediately prior to Fontan. To isolate the effect of BDCPC on ventricular function, only patients with normal EF at baseline, defined as EF ≥ 50%, were considered for the primary endpoints. The primary endpoints were defined by the mean change in visually estimated EF at hospital discharge and the mean EF evolution over time from BDCPC to Fontan, according to ventricular morphology.

To assess the reliability of our 2D echocardiographic visually estimated EF measurement, one author (MV) performed a second blinded analysis, on a subset of 30 patients randomly selected at each available time-point.

We finally compared EF results obtained by 2D echocardiography (visually estimated EF) and by catheterization (volumetric EF) at pre-BDCPC and pre-Fontan time-points.

### Survival Analysis

The most recent cardiovascular clinical update was collected to assess long-term prognosis. Major cardiovascular events included the following: Fontan circulation failure, Fontan or BDCPC takedown, not a candidate for Fontan completion, thromboembolism, protein-losing enteropathy, plastic bronchitis, liver cirrhosis, heart transplantation, or death. A freedom from major cardiac event survival curve was constructed for each group according to ventricle morphology and discharge EF.

### Risk Factor Analysis

Evaluation of risk factors for a major cardiac event was performed to investigate long-term prognosis based on the previous results. Factors that were studied included ventricular dysfunction pre-BDCPC, ventricular dysfunction post-BDCPC (hospital discharge), RV morphology, HLHS anatomy, age at BDCPC < 4 months old, extra cardiac disease, BDCPC bypass time > 65 min, and Nakata index pre-BDCPC < 200 mm/m^2^. EF < 50% was defined as ventricular dysfunction. The secondary endpoint was defined by the hazard ratio (HR) for major cardiac event in patients with ventricular dysfunction post-BDCPC adjusted to baseline EF.

### Statistical Analysis

Comparative analysis was conducted between the LV group and the entire RV group.

To isolate the effect of HLHS morphology, a subgroup analysis was performed dividing the RV group into HLHS group and non-HLHS RV group and another comparative analysis was conducted among the LV group, the HLHS group, and the non-HLHS RV group.

Descriptive statistics included calculations of mean and standard deviations or median and interquartile 1, 3 for continuous variables according to their distribution, counts, and percent for categorical variables. Differences between groups according to descriptive parameters and mean EF changes (primary endpoint) were analyzed using t-test or one-way ANOVA. Repeated-measures analysis using proc mixed in was performed to show differences in mean EF evolution between groups over time.

Inter-reader reliability for 2D echo visually estimated EF measurement was evaluated using intra-class correlation coefficients (ICC) [16]. ICC values less than 0.5, between 0.5 and 0.75, between 0.75 and 0.9, and greater than 0.9 were indicative of poor, moderate, good, and excellent reliability, respectively [17]. Bland–Altman analysis [12], Spearman's correlation and linear regression were used to compare EF values obtained by 2D echo (visual assessment) and by catheterization (volumetric calculation). Survival models and risk factor analysis were employed using proc phreg SAS. We performed an adjusted survival analysis of time from BDCPC to the major cardiac events previously described.

Analyses were performed using GraphPad Prism version 5.04 (GraphPad software, San Diego, CA) and SAS software version 9.4 (SAS Institute Inc.Cary, NC). A *p* value < 0.05 was considered statistically significant.

## Results

Ninety-two patients met the inclusion criteria in this study. 52 had LV morphology and 40 had RV morphology (28/40 had HLHS and 12/40 had non-HLHS RV). Specific cardiac lesions included HLHS (30%), tricuspid atresia (22%), double outlet right ventricle (12%), pulmonary atresia with intact ventricular septum (10%), double inlet left ventricle (9%), and unbalanced atrioventricular canal (9%).

Table 1 summarizes descriptive data pre-BDCPC. The RV group had a BDCPC at a younger age than the LV group (6.6 ± 4.3 mos. vs 9.7 ± 9.6 mos., respectively, *p* = 0.045). The HLHS group (5.3 ± 1.4 mos.) was the youngest at time of BDCPC. The Nakata index was significantly smaller in the RV group than in the LV group (184 ± 94 mm/m^2^ vs. 251 ± 125 mm/m^2^, respectively, *p* = 0.003). In the HLHS group, the Nakata index was the lowest (162 ± 84 mm/m^2^).

**Table 1:** Pre-BDCPC data

Characteristics	Total (*N* = 92)	LV group (*N* = 52)	RV group (*N* = 40)	HLHS group (*N* = 28)	Non-HLHS RV group (*N* = 12)	*p* value Comparison LV *vs.* RV	*p* value Comparison LV *vs.* HLHS *vs* non-HLHS RV
*Demographic data* Age (months)	8.3 (7.8)	9.7 (9.6)	6.6 (4.3)	5.3 (1.4)	9.8 (6.9)	**0.045**	0.064
Weight (kg)	7.0 (2.1)	7.2 (2.3)	6.8 (1.7)	6.3 (1.6)	7.8 (1.4)	0.063	0.334
Height (cm)	65.7 (8.2)	66.4 (8.9)	64.8 (7.2)	62.2 (4.1)	71.1 (8.9)	**0.004**	0.385
BSA (m^2^)	0.36 (0.07)	0.37 (0.08)	0.35 (0.05)	0.33 (0.04)	0.40 (0.06)	**0.01**	0.319
Male *N* (%)	50 (54.3)	29 (55.7)	21(52.5)	12 (42.9)	9 (75.0)	0.166	0.834
Oxygen Saturation (%) Hb (g/dl)	75 (7) 15.1 (2.3)	75 (7) 15.2 (2.6)	76 (7) 15.0 (2.0)	76 (7) 14.7 (2.0)	76 (7) 15.7 (1.7)	0.942 0.471	0.754 0.687
*Extracardiac disease* *N* (%)							
None	60 (65.2)	39 (75.0)	21 (52.5)	15 (53.6)	6 (50.0)	**0.029**	0.078
Heterotaxy	10 (10.8)	5 (9.6)	5 (12.5)	0 (0)	5 (41.6)	0.742	** < 0.001**
Syndromic disease	9 (9.8)	4 (7.7)	5 (12.5)	5 (17.8)	0 (0)	0.495	0.163
Other severe chronic disease	13 (14.1)	4 (7.7)	9 (22.5)	8 (28.6)	1 (8.3)	0.068	**0.031**
Bilateral SVC *N* (%)	11 (11.9)	2 (3.8)	9 (22.5)	4 (14.3)	5 (41.7)	**0.008**	**0.001**
*Previous surgery* *N* (%) None	12 (13.0)	7 (13.5)	5 (12.5)	0 (0)	5 (41.7)	> 0.99	**0.002**
Norwood-Blalock shunt	11 (12.0)	6 (11.5)	5 (12.5)	5 (17.8)	0 (0)	> 0.99	0.277
Norwood-Sano shunt	24 (26.1)	2 (3.8)	22 (55.0)	22 (78.6)	0 (0)	** < 0.001**	** < 0.001**
Norwood-hybrid	1 (1.1)	0 (0)	1 (2.5)	1 (3.6)	0 (0)	0.435	0.315
Systemic-pulmonary shunt	34 (37.0)	29 (56.0)	5 (12.5)	0 (0)	5 (41.7)	** < 0.001**	** < 0.001**
PA band Other	8 (8.7) 2 (2.2)	7 (13.5) 1 (1.9)	1 (2.5) 1 (2.5)	0 (0) 0 (0)	1 (8.3) 1 (8.3)	0.132 > 0.99	0.252 0.249
*Catheterization pre-BDCPC* Total *N* (%)	73/87 (90.8%)	38/49 (77.6%)	35/38 (92.1)	26/28 (92.9%)	9/10 (90%)	0.083	0.182
Interventional *N* (%) Mean PAP (mmHg)	11/73 (15.1%) 14 (4)	3/38 (7.9%) 14 (3)	8/35 (22.9%) 14 (5)	7/26 (26.9%) 15 (5)	1/9 (11.1%) 13 (3)	0.106 0.311	0.104 0.932
TP gradient (mmHg)	7 (3)	7 (3)	7 (4)	8 (4)	6 (3)	0.294	0.879
SVEDP (mmHg)	9 (2)	9 (2)	9 (2)	9 (2)	8 (2)	0.248	0.113
Nakata index (mm/m^2^)	221 (115)	251 (125)	184 (94)	162 (84)	274 (83)	**0.003**	0.054
Qp:Qs ratio	1 (0.7)	1 (0.6)	1.1 (0.70)	1.1 (0.8)	1.2 (0.6)	0.721	0.469
Volumetric EF (%)	57 (9)	61 (9)	56 (8)	55 (10)	59 (9)	**0.02**	**0.036**
*Medication profile pre-BDCPC* *N* (%)							
None	16 (17.4)	9 (17.3)	7 (17.5)	1 (3.6)	6 (50.0)	> 0.99	**0.002**
Aspirin	45 (48.9)	25 (48.1)	20 (50.0)	19 (67.9)	1 (8.3)	> 0.99	**0.003**
Diuretics	28 (30.4)	13 (25.0)	15 (37.5)	13 (46.4)	2 (16.7)	0.254	0.075
Digoxin	30 (32.6)	14 (26.9)	16 (40.0)	13 (46.4)	3 (25.0)	0.262	0.173
ACE inhibitor	14 (15.2)	4 (7.7)	10 (25.0)	10 (35.7)	0 (0)	**0.038**	**0.001**

Results are in mean (standard deviation) and N (%). Bold indicates significant values

*BDCPC* bidirectional cavopulmonary connection, *LV* left ventricle, *RV* right ventricle, *HLHS* hypoplastic left heart syndrome, *BSA* body surface area, *Hb* hemoglobin, chrs chromosomic, *SVC* superior vena cava, *PA* pulmonary artery,* PAP* pulmonary artery pressure, *TP* transpulmonary pressure, SVEDP single ventricle end diastolic pressure, *QP* Pulmonary flow, *QS* Systemic flow, *EF* Ejection Fraction, *ACE* angiotensin conversion enzyme.

Table 2 summarized descriptive data at time of BDCPC. Almost all (92%) patients had BDCPC using cardiopulmonary bypass and 68.5% underwent an additional procedure during BDCPC. No in-hospital complications were reported for 63% of the patients. One patient died before hospital discharge, at 6 days post-operative, due to hemodynamic failure (this patient came to BDCPC with an LV dominant unbalanced AV canal, severe AV valve regurgitation, and ventricular dysfunction). One patient had BDCPC takedown. That patient had HLHS with a small left superior vena cava and an occluded right superior vena cava who needed a systemic-pulmonary artery shunt intra-operatively.

**Table 2:** BDCPC: operative and post-operative data

Variables	Total (*N* = 92)	LV group (*N* = 52)	RV group (*N* = 40)	HLHS group (*N* = 28)	Non-HLHS RV group (*N* = 12)	*p* value Comparison LV vs. RV	*p* value Comparison LV vs. HLHS vs non-HLHS RV
*Cardiopulmonary bypass time* *N* (%) Time (min)	85 (92.4) 65 [42;92]	47 (90.4) 65 [45;101]	38 (95.0) 65 [42;87]	26 (92.8) 63 [40;95]	12 (100) 67 [49;83]	0.163	0.575
*Circulatory arrest time* *N* (%) Time (min)	39 (42.4) 21 [16;31]	25 (48.1) 23 [16.5;35]	14 (35.0) 19 [14.5;22.5]	9 (32.1) 19 [14;24]	5 (41.7) 20 [15.5;21.5]	0.287 0.169	0.387 0.385
Deep hypothermic circulatory arrest < 28 °C N (%)	39 (42.3)	19 (36.5)	20 (50.0)	13 (46.4)	7 (58.3)	0.062	0.339
*Additional surgical procedure* *N* (%)							
None	29 (31.5)	16 (30.8)	13 (32.5)	8 (28.6)	5 (41.7)	> 0.99	0.705
Atrioventricular valve repair	8 (8.6)	5 (9.6)	3 (7.5)	2 (7.1)	1 (8.3)	> 0.99	0.931
Pulmonary arterioplasty	33 (35.8)	18 (34.6)	15 (37.5)	13 (46.4)	2 (16.7)	0.828	0.191
Aortic arch repair	7 (7.6)	2 (3.8)	5 (12.5)	5 (17.9)	0 (0)	0.233	**0.045**
Atrial septectomy	15 (16.3)	8 (15.4)	7 (17.5)	6 (21.4)	1 (8.3)	0.746	0.568
Other	25 (27.2)	17 (32.7)	8 (20.0)	5 (17.9)	3 (25.0)	0.238	0.358
Peak post-op lactate levels (mmol/L)	2.25 [1.6;3.4]	2 [1.3;2.85]	2.8 [1.8;4.7]	3.0 [2.0;4.9]	1.8 [1.5;2.6]	0.068	0.069
Hospital length of stay (days)	7 [6;9]	6 [5;9]	8 [6;10]	8.5 [6;15.5]	7.5 [6;8]	0.218	0.127
Post-op inhaled NO use *N* (%)	15 (16.3)	8 (15.4)	7 (18.4)	7 (25.0)	0 (0)	0.785	0.141
Duration of intubation (days)	1 [0.5;3]	1 [0.5;3]	1 [0.5;2.8]	1 [0.5;3]	1 [0.6;1]	0.167	0.065
*Complications prior to discharge* *N* (%)							
none	58 (63.0)	34 (65.4)	24 (60)	17 (60.7)	7 (58.3)	0.666	0.859
respiratory	15 (16.3)	7 (13.5)	8 (20.5)	5 (17.9)	3 (25.0)	0.411	0.599
infectious	10 (10.9)	5 (9.6)	5 (12.8)	5 (17.9)	0 (0)	0.742	0.228
hemodynamic instability	11 (12.0)	6 (11.5)	5 (12.8)	4 (14.3)	1 (8.3)	> 0.99	0.859
gastrointestinal	5 (5.4)	1 (1.9)	4 (10.3)	4 (14.3)	0 (0)	0.163	0.445
death	1 (1.1)	1 (1.9)	0 (0)	0 (0)	0 (0)	> 0.99	0.678
Creatinine at discharge (mg/dl)	0.3 [0.2;0.4]	0.3 [0.2;0.4]	0.4 [0.2;0.5]	0.3 [0.2;0.4]	0.4 [0.3;0.5]	0.588	0.331
*Medication profile at discharge* *N* (%)							
None	1 (10.9)	1 (1.9)	0 (0)	0 (0)	0 (0)	> 0.999	0.672
Aspirin	71 (78.90	41 (80.4)	34 (85.0)	25 (89.3)	9 (75.0)	0.782	0.469
Diuretics	78 (85.7)	41 (80.4)	37 (92.5)	27 (96.4)	10 (83.3)	0.248	0.145
Digoxin	33 (36.2)	11 (21.6)	22 (55.5)	16 (57.1)	6 (50.0)	**0.002**	**0.004**
ACE inhibitor	36 (39.6)	18 (35.3)	18 (45.0)	12 (42.8)	6 (50.0)	0.392	0.587
PAH treatment	3 (3.3)	1 (1.9)	2 (5)	2 (7.1)	0 (0)	0.580	0.406
*Catheterization pre-Fontan*							
Total *N* (%)	64/70 (91.4)	35/39 (89.7)	29/31 (93.5)	19/20 (95.0)	10/11 (90.9)	0.684	0.776
Mean PAP (mmHg)	13 (2)	12 (2)	13 (2)	12 (2)	14 (2)	0.761	0.107
TP gradient (mmHg)	5 (2)	5 (2)	4 (2)	4 (1)	5 (3)	0.143	0.137
SVEDP (mmHg)	10 (7)	11 (9)	9 (2)	9 (2)	9 (3)	0.198	0.441
Volumetric EF (%)	57 (8)	59 (7)	54 (8)	53 (8)	55 (9)	0.061	0.169

Results are in median [interquartile 1;3] or N (%).Bold indicates significant values

*BDCPC* bidirectional cavopulmonary connection, *LV* left ventricle, *RV* right ventricle, *HLHS* hypoplastic left heart syndrome, *NO* nitric oxide, *ACE* angiotensin conversion enzyme, *PAH* pulmonary artery hypertension, *EF* ejection fraction

Eleven of 92 patients (16%) had a > Grade 1 AV valve regurgitation pre-BDCPC. Eight of these 11 patients underwent AV valve surgical repair at time of BDCPC. 7 patients still had a > Grade 1 AV valve regurgitation at time of Fontan. In the non-HLHS RV group, AV valve function declined with time: 8% patients had > Grade 1 regurgitation at pre-BDCPC while 27% had > Grade 1 regurgitation prior to Fontan. There was a significant difference between groups in > Grade 1 AV valve regurgitation at pre-Fontan evaluation (5% in LV group, 10% in HLHS group and 27% in non-HLHS RV group, *p* = 0.04). Five of 92 patients (5%), all with HLHS, had > Grade 1 aortic arch obstruction pre-stage II. Four of these 5 patients had aortic arch enlargement at time of BDCPC. None had > grade 1 aortic arch obstruction at time of Fontan.

### Ventricular Function Analysis

Table 3 reports 2D echo visually estimated EF results from the entire cohort. At baseline, EF mean value was significantly lower in the HLHS group vs the non-HLHS RV and LV groups (55 ± 7%, vs 58 ± 3% and 61 ± 5%, respectively, *p* < 0.01). At discharge, there was a significant decrease of mean EF in the HLHS group (55% to 49 ± 7% (− 6%), *p* < 0.01) and RV non-HLHS group (58 ± 3% to 54 ± 6% (− 4%), *p* = 0.04) while EF remained stable in the LV group (61 ± 5% to 59% (− 2%), *p* = 0.19). In the HLHS group, EF did not recover pre-Fontan as compared to the non-HLHS RV and LV groups (53 ± 3% vs 56 ± 6% and 60 ± 4%, *p* < 0.01).

**Table 3:** Mean EF evolution according to ventricular morphology in the entire cohort

Time of analysis	Total	LV group	RV group	HLHS group	Non-HLHS RV group	*p* value Comparison LV vs. RV	*p* value Comparison LV vs. HLHS vs non-HLHS RV
*Pre-BDCPC*	59 ± 6% (*N* = 92)	61 ± 5% (*N* = 52)	56 ± 6% (*N* = 40)	55 ± 7% (*N* = 28)	58 ± 3% (*N* = 12)	** < 0.01**	** < 0.01**
*Hospital discharge*	55 ± 7% (*N* = 92)	59 ± 6% (*N* = 52)	50 ± 7% (*N* = 40)	49 ± 7% (*N* = 28)	54 ± 6% (*N* = 12)	** < 0.01**	** < 0.01**
*1–3 months post-BDCPC*	57 ± 6% (*N* = 42)	59 ± 4% (*N* = 21)	52 ± 5% (*N* = 21)	51 ± 5% (*N* = 17)	56 ± 3% (*N* = 4)	** < 0.01**	** < 0.01**
*6–12 months post-BDCPC*	56 ± 7% (*N* = 66)	59 ± 5% (*N* = 33)	51 ± 7% (*N* = 33)	50 ± 7% (*N* = 26)	56 ± 6% (*N* = 7)	** < 0.01**	** < 0.01**
*Pre-Fontan*	58 ± 5% (*N* = 69)	60 ± 4% (*N* = 39)	54 ± 4% (*N* = 30)	53 ± 3% (*N* = 19)	56 ± 6% (*N* = 10)	** < 0.01**	** < 0.01**

Results are in mean ± standard deviation. Bold indicates significant values

*BDCPC* bidirectional cavopulmonary connection, *EF* ejection fraction, *LV* left ventricle, *RV* right ventricle, *HLHS* hypoplastic left heart syndrome

Table 4 reports 2D echo visually estimated EF results for patients with EF ≥ 50% at baseline. Results were similar, but the decrease from baseline to discharge was larger in the HLHS group: 59 ± 4% to 49 ± 7% in the HLHS group vs. 58 ± 3% to 54 ± 6% in the non-HLHS RV group and 61 ± 4% to 60 ± 4% in the LV group. Therefore, a mean EF decrease of 9% (*p* < 0.01) vs 4% (*p* = 0.04) and 1% (*p* = 0.14), respectively.

**Table 4:** Mean EF evolution according to ventricular morphology for patients with EF ≥ 50% at baseline

Time of analysis	Total	LV group	RV group	HLHS group	Non-HLHS RV group	*p* value Comparison LV vs. RV	*p* value Comparison LV vs. HLHS vs non-HLHS RV
Pre-BDCPC	60 ± 4% (*N* = 81)	61 ± 4% (*N* = 49)	58 ± 3% (*N* = 32)	59 ± 4% (*N* = 20)	58 ± 3% (*N* = 12)	** < 0.01**	**0.02**
Hospital discharge	56 ± 7% (*N* = 81)	60 ± 4% (*N* = 49)	51 ± 7% (*N* = 32)	49 ± 7% (*N* = 20)	54 ± 6% (*N* = 12)	** < 0.01**	** < 0.01**
1–3 months post-BDCPC	56 ± 5% (*N* = 33)	59 ± 4% (*N* = 18)	52 ± 4% (*N* = 15)	51 ± 4% (*N* = 11)	56 ± 3% (*N* = 4)	** < 0.01**	** < 0.01**
6–12 months post-BDCPC	56 ± 6% (*N* = 56)	59 ± 5% (*N* = 31)	52 ± 7% (*N* = 25)	50 ± 7% (*N* = 18)	56 ± 6% (*N* = 7)	** < 0.01**	** < 0.01**
Pre-Fontan	58 ± 5% (*N* = 64)	60 ± 4% (*N* = 39)	55 ± 4% (*N* = 25)	54 ± 2% (*N* = 14)	56 ± 6% (*N* = 11)	** < 0.01**	** < 0.01**

Results are in mean ± standard deviation. Bold indicates significant values

*BDCPC* bidirectional cavopulmonary connection, *EF* ejection fraction, *LV* left ventricle, *RV* right ventricle, *HLHS* hypoplastic left heart syndrome

**Fig. 1:**
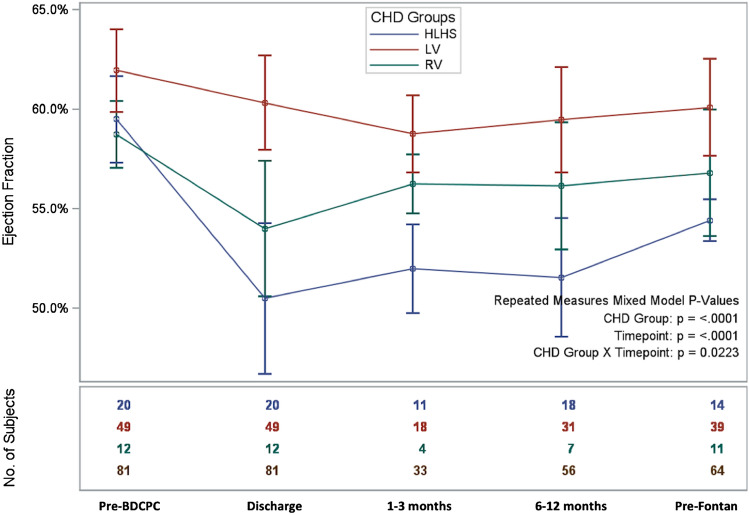
Ejection fraction evolution after BDCPC according to single ventricle morphology, patients with EF ≥ 50% pre-BDCPC. This line graph represents the evolution over time of the 2D echo visually estimated EF for patients with EF ≥ 50% at baseline according to ventricular morphology (red line = LV group, green line = non-HLHS RV group, blue line = HLHS group). Repeated-measures analysis using proc mixed in SAS software version 9.4 (SAS Institute Inc., Cary, NC) was performed to show differences in mean EF evolution between groups over time. There was a significant difference between groups over time, with a more poor evolution from pre-BDCPC to pre-Fontan in the HLHS group than in the non-HLHS RV and LV groups (*p* = 0.02). *BDCPC* bidirectional cavopulmonary connection, *CHD* congenital heart disease, *HLHS* Hypoplastic Left Heart Syndrome, *LV* Left ventricle, *RV* non-HLHS RV

Figure 1 represents the evolution over time of 2D echo visually estimated EF for patients with EF ≥ 50% at baseline according to ventricular morphology. There was a significant difference between groups over time, with a more poor evolution in the HLHS group than in the non-HLHS RV and LV groups (*p* = 0.02).

2D echo visually estimated EF measurement was associated with good inter-reader reliability: ICC = 0.88; 95% CI [0.75–0.95]. The comparison between EF values obtained by 2D echo and catheterization at pre-BDCPC and pre-Fontan time-points also showed a good reliability with a mean error (bias) of 1.3%; 95% CI [− 5%– + 7%] and a Spearman’s coefficient rho = 0.64, *p* < 0.0001. We found a linear correlation between those two methods: *Y* = 0.91 × *X* + 0.03 (*p* < 0.0001).

### Survival Analysis

The median follow-up was 8 years [4;12] after BDCPC, there was no difference between groups (8 years [3;11] for the LV group and 7 years [4;14] for the RV group, *p* = 0.36). Thirty-five patients (19 in the LV group and 16 in the RV group) had ≥ 10 years follow-up. Seventy-seven percent of patients underwent Fontan at a median age of 34 months [30;40] with an interval between BDCPC and Fontan procedures of 26 months [21;34]. One patient was lost to follow-up, and therefore included as censured data in the survival analysis.

Thirteen major cardiac events occurred, mainly in the RV group (17.5% vs 9.6%, *p* = 0.35). Most patients with a major cardiac event (62%) experienced ventricular dysfunction post-BDCPC (EF < 50% at hospital discharge). Major cardiac events were reported as follows:

- 6 patients had Fontan circulation failure or were not Fontan candidates
  1 patient had BDCPC takedown (detailed above), 1 patient had Fontan takedown (due to pulmonary vein stenosis), 2 patients were not candidates for Fontan procedure because of pulmonary artery hypoplasia, and 2 others were considered as “failing Fontan” (due to reduced EF and extracardiac conduit thrombosis in one patient, and severe AV regurgitation with cyanosis in the other patient). Four of these 6 patients experienced ventricular dysfunction post-BDCPC.
- 4 patients died: 1 after BDCPC (detailed above) and 3 after Fontan (all with HLHS, 1 from RSV bronchiolitis, 1 from Pseudomonas aeruginosa pneumonia and 1 from acidosis due to lower extremity ischemia). All patients experienced ventricular dysfunction post-BDCPC.
- 3 patients had heart transplantation at 14 [12;16] years old; 11 [9;13.5] years post-Fontan, all were in the RV group and none had ventricular dysfunction post-BDCPC.

**Fig. 2:**
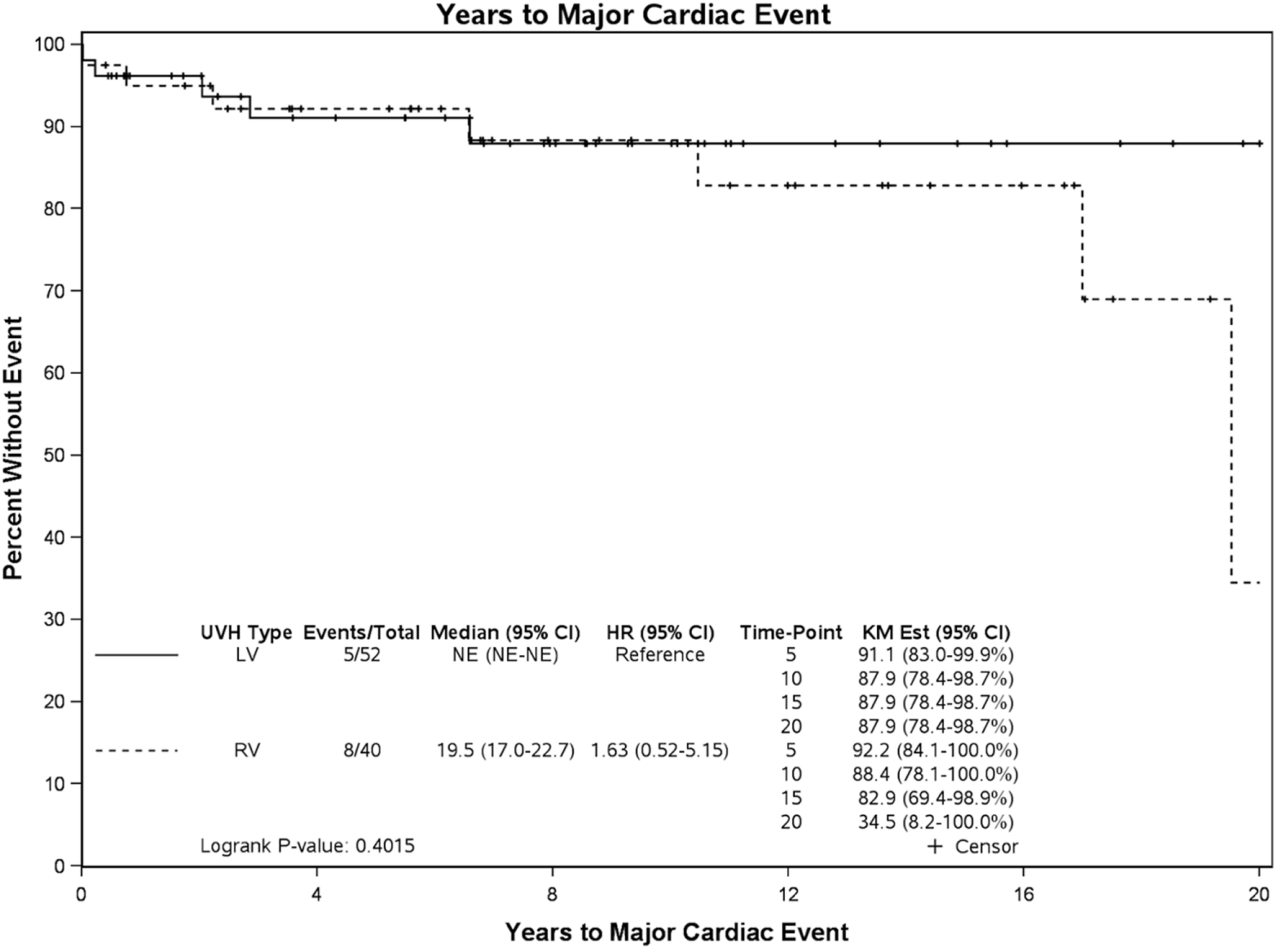
Major cardiac survival curve according to single ventricle morphology. *UVH* Univentricular Heart, i.e., single ventricle morphology, *LV* Left Ventricle, *RV* Right Ventricle

**Fig. 3:**
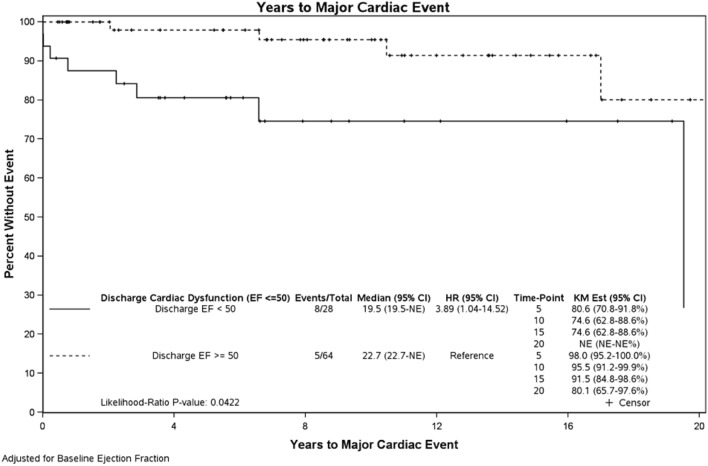
Major cardiac survival curve according to discharge EF. *EF* Ejection Fraction

Figures 2 and 3 show major cardiac event survival curves: RV patients had a higher hazard ratio (HR) (1.63; 95% CI (0.52, 5.15)) than LV patients, with 35% vs. 88% freedom from major cardiac event at 20 years, respectively. This difference was not statistically significant (*p* = 0.40). Patients with post-BDCPC ventricular dysfunction (discharge EF < 50%) had a higher HR (HR 3.89; 95% Cl 1.04–14.52) than those with a discharge EF ≥ 50%, with 75% vs. 92% freedom from major cardiac events at 15 years, respectively, (*p* = 0.04).

### Risk Factor Analysis

The HR for major cardiac events in patients with pre-BDCPC ventricular dysfunction (pre-BDCPC EF < 50%) is 7.8 times higher than for patients with pre-BDCPC EF of ≥ 50% (*p* < 0.001). The HR for major cardiac event in patients with post-BDCPC ventricular dysfunction (discharge EF < 50%, adjusted on pre-BDCPC EF) is 3.9 times higher than patients with a discharge EF of ≥ 50% (*p* = 0.04). Every 5% decrease in EF from baseline to discharge was associated with a 1.63 HR. This result approached statistical significance (*p* = 0.052). Younger age at BDCPC (< 4 months old) and associated extracardiac disease were also associated with a higher HR of a major cardiac event. Finally, we found no significant association between Nakata index, cardiopulmonary bypass time, or ventricular morphology with major cardiac events (Table 5).

**Table 5:** Risk factor analysis

Hazard Ratios for Major Cardiac Event			
Description	HR	95% confidence limits	
RV morphology	1.64	0.52	5.26
HLHS morphology	1.34	0.39	4.59
Nakata index < 200 mm/m^2^	1.22	0.11	13.5
Age at BDCPC < 4 months old	**4.28**	**1.29**	**14.19**
Extracardiac disease*	**3.57**	**1.11**	**11.11**
Bypass time > 65 min	1.27	0.40	4.0
Pre-BDCPC EF < 50%	**7.80**	**2.17**	**28.03**
Discharge EF < 50% (adjusted for pre-BDCPC EF)	**3.89**	**1.04**	**14.52**
> 5% change in EF from pre-BDCPC to discharge	1.627	0.996	2.659

Bold indicates significant values

*BDCPC* bidirectional cavopulmonary connection, *EF* ejection fraction, *LV* left ventricle, *RV* right ventricle, *HLHS* hypoplastic left heart syndrome, *HR* hazard ratio

*Heterotaxy, syndromic disease or other severe chronic disease

## Discussion

We conducted the first functional SV study investigating the period between BDCPC and Fontan procedures using EF evaluation. Our first goal was to define whether the BDCPC impacted the SV function according to ventricular morphology. We found a decrease in the mean EF in the RV group after BDCPC, especially in the HLHS subset. This 9% drop in the HLHS group at hospital discharge was clinically relevant, and these patients did not completely recover function before Fontan. In contrast, the mean EF in the LV group remained stable over time.

Several studies have shown a decrease in single RV contractility at different stages of palliation with standard echo parameters [13], 2D strain [18–20], 3D real-time echo [21], or cardiac MRI [22]. Kutty et al. showed an 8% decrease in EF with 3D real-time echo (46 ± 5% before and 38 ± 4% after BDCPC) in patients with HLHS, which is comparable to our results [21].

The etiology of decreased contractility post-BDCPC in the patient with functional single RV remains unclear. The role of loading changes, including decreased preload [21] and chronic pressure overload [23] on an inadequate ventricle, is the most frequent explanation. Embryologic, histologic, and anatomic differences between the RV and LV seem to impact response to load variation, despite RV remodeling [23]. Diffuse fibrosis on cardiac MRI is reported after Fontan in patients with RV morphology compared to LV [24]. 2D strain studies in single RV patients showed that changes in circumferential contraction over time: decreased circumferential stain after stage I [18, 25] and reduced longitudinal to circumferential ratio after stage III [19], mimic changes in LV contraction. This may result in lower coronary flow reserve [26] and a potentially greater influence of ischemia due to recurrent surgeries.

Furthermore, mechanical dyssynchrony, if not the major factor explaining early cardiac dysfunction, may impact RV function in older patients [27, 28]. Finally, tricuspid regurgitation due to progressive RV dilatation contributes to worsening of ventricular function [29]. Consistently in our study, AV valve dysfunction was more common in the RV group than in the LV group.

Why does ventricular function become worse in the HLHS subgroup after stage II palliation? In addition to the RV morphology, some other negative factors have been previously linked to HLHS anatomy: complex stage I palliation with high post-operative morbidity [30], younger age at stage II [31], recurrent aortic arch obstruction [32], and pulmonary artery hypoplasia [33]. In our study, the HLHS group had all of these factors which potentially explained its great vulnerability.

Our second objective was to determine if the decrease in ventricular function after BDCPC was associated with poor long-term prognosis. We found that EF < 50% at BDCPC discharge was strongly correlated with a higher risk (3.9×) for major cardiac events including Fontan circulation failure, heart transplantation, or death. Similarly, the risk of a major cardiac event was proportional to the post-BDCPC EF decrease.

Retrospective studies of patients with functional SV with long-term follow-up data reported other risk factors that negatively impact prognosis including arrhythmia, atrioventricular valve regurgitation, and prolonged pleural effusion drainage [6, 34]. Two large consortium studies (Single Ventricle Reconstruction Trial from North America [4, 5, 35] and the Australian and New Zealand Stage III Registry [8]) followed patients with functional SV physiology over time. In these studies, atrioventricular valve regurgitation and heterotaxy were predictors of mortality [8]. But source of pulmonary blood flow (modified Blalock-Taussig shunt versus RV to the pulmonary artery conduit (Sano shunt)) at stage I did not change long-term prognosis [4, 5, 35].

Several studies have correlated SV function to prognosis: qualitative SV dysfunction pre-Norwood [36], lower longitudinal and circumferential strain in the interstage period [37], and smaller tissue Doppler annular displacement pre-Stage II [38]. These issues have been associated with a shorter transplant-free survival in patients with HLHS.

Similar to other reports [6, 39], our study also did not demonstrate a correlation between SV morphology and long-term prognosis. However, HLHS anatomy is known to be associated with a more poor long-term prognosis [8, 40] and the follow-up of this group is still limited.

HLHS anatomy in our study was strongly associated with post-BDCPC ventricular dysfunction which was related to poor long-term prognosis. Despite improvements in surgical techniques and anesthesia management, medical treatment of RV failure remains ineffective [41, 42] and heart transplantation remains the definitive therapy for end-stage RV failure. Preservation of RV function should be a major goal at each step of palliation for patients with HLHS. Hopefully, novel therapies may alter this natural history.

### Limitations

Mayo Clinic is a tertiary care referral center for congenital heart disease. This study is limited by the number of patients who had follow-up echocardiograms at our institution.

Assessing functionally SV function remains an unresolved issue especially for RV type morphology. Since this was a retrospective study, biplane pyramidal EF, strain analysis, 3D echocardiography, and cardiac MRI were not available for all patients. We also needed imaging data pre- and post-stage II. For now, only standard 2D echocardiography is considered as appropriate imaging for routine surveillance post-stage II [43]; hence, reliance on 2D echocardiography until cardiac MRI pre- and post-stage II becomes standard of care. Furthermore, we wanted to use one common parameter for LV and RV morphology in order to compare the evolution of ventricular function in the LV and RV groups with the same contractility parameter (EF) and therefore similar limitations regarding dependency on load conditions. We found in fact that the EF decline was much worse in the RV group (especially for patients with HLHS) than in the LV group. Visually estimated EF is a subjective evaluation of ventricular contractility but its reliability to objective parameters (automated biplane EF for LV and MRI for RV) have been reported [14, 15] and in our study, volumetric EF measurements from catheterization at pre-BDCPC and pre-Fontan time were similar and correlated to the same time-point echocardiographic values. Finally, our 5% decrease in EF used in the risk factor analysis may be within the margin of error of the estimated EF.

Major cardiac event survival curves according to SV morphology showed no difference until 10 years post-stage II follow-up and then a separation of the two curves with big steps down for the RV group but without significant difference between groups. Small number of patients with long-term follow-up may be confounding factor and potentially explains why the difference between groups was not statistically significant.

A prospective study is critically needed given these limitations.

## Conclusion

Compared to patients with LV morphology, patients with RV morphology, especially those with HLHS, are at greater risk of post-BDCPC ventricular dysfunction. This dysfunction is associated with long-term major cardiac events. Strategies to maintain ventricular function as normal as possible at BDCPC could be reasonably expected to contribute to improved long-term prognosis, especially for patients with HLHS. These results should be confirmed by a multicenter prospective study to minimize inclusion bias. Preserving ventricular function remains a therapeutic challenge for the care team. New therapeutic approaches are needed in this regard.

## References

[1] Herrmann JL, Brown JW (2019). The superior cavopulmonary connection: history and current perspectives. World J Pediatr Congenit Heart Surg.

[2] Kim AS, Witzenburg CM, Conaway M (2019). Trajectory of right ventricular indices is an early predictor of outcomes in hypoplastic left heart syndrome. Congenit Heart Dis.

[3] Simsic JM, Phelps C, Kirchner K (2018). Interstage outcomes in single ventricle patients undergoing hybrid stage 1 palliation. Congenit Heart Dis.

[4] Tabbutt S, Ghanayem N, Ravishankar C (2012). Risk factors for hospital morbidity and mortality after the Norwood procedure: a report from the pediatric heart network single ventricle reconstruction trial. J Thorac Cardiovasc Surg.

[5] Ghanayem NS, Allen KR, Tabbutt S (2012). Interstage mortality after the Norwood procedure: results of the multicenter single ventricle reconstruction trial. J Thorac Cardiovasc Surg.

[6] Pundi KN, Johnson JN, Dearani JA (2015). 40-Year follow-up after the Fontan operation: long-term outcomes of 1,052 patients. J Am Coll Cardiol.

[7] Khairy P, Fernandes SM, Mayer JE (2008). Long-term survival, modes of death, and predictors of mortality in patients with Fontan surgery. Circulation.

[8] d’Udekem Y, Iyengar AJ, Galati JC (2014). Redefining expectations of long-term survival after the Fontan procedure: twenty-five years of follow-up from the entire population of Australia and New Zealand. Circulation.

[9] Eckhauser A, Pasquali SK, Ravishankar C (2018). Variation in care for infants undergoing the Stage II palliation for hypoplastic left heart syndrome. Cardiol Young.

[10] Di Molfetta A, Iacobelli R, Guccione P (2017). Evolution of ventricular energetics in the different stages of palliation of hypoplastic left heart syndrome: a retrospective clinical study. Pediatr Cardiol.

[11] Arnold RR, Loukanov T, Gorenflo M (2014). Hypoplastic left heart syndrome-unresolved issues. Front Pediatr.

[12] Bland JM, Altman DG (1986). Statistical methods for assessing agreement between two methods of clinical measurement. Lancet Lond Engl.

[13] Mahle WT, Coon PD, Wernovsky G, Rychik J (2001). Quantitative echocardiographic assessment of the performance of the functionally single right ventricle after the Fontan operation. Cardiol Young.

[14] Abazid RM, Abohamr SI, Smettei OA (2018). Visual versus fully automated assessment of left ventricular ejection fraction. Avicenna J Med.

[15] Schneider M, Ran H, Aschauer S (2019). Visual assessment of right ventricular function by echocardiography: how good are we?. Int J Cardiovasc Imaging.

[16] Shrout PE, Fleiss JL (1979). Intraclass correlations: uses in assessing rater reliability. Psychol Bull.

[17] Koo TK, Li MY (2016). A guideline of selecting and reporting intraclass correlation coefficients for reliability research. J Chiropr Med.

[18] Kaneko S, Khoo NS, Smallhorn JF, Tham EB (2012). Single right ventricles have impaired systolic and diastolic function compared to those of left ventricular morphology. J Am Soc Echocardiogr.

[19] Tham EB, Smallhorn JF, Kaneko S (2014). Insights into the evolution of myocardial dysfunction in the functionally single right ventricle between staged palliations using speckle-tracking echocardiography. J Am Soc Echocardiogr.

[20] Suntratonpipat S, Khoo NS, Colen T (2017). Impaired single right ventricular function compared to single left ventricles during the early stages of palliation: a longitudinal study. J Am Soc Echocardiogr.

[21] Kutty S, Graney BA, Khoo NS (2012). Serial assessment of right ventricular volume and function in surgically palliated hypoplastic left heart syndrome using real-time transthoracic three-dimensional echocardiography. J Am Soc Echocardiogr.

[22] Sundareswaran KS, Kanter KR, Kitajima HD (2006). Impaired power output and cardiac index with hypoplastic left heart syndrome: a magnetic resonance imaging study. Ann Thorac Surg.

[23] Si M-S, Ohye RG (2017). Stem cell therapy for the systemic right ventricle. Expert Rev Cardiovasc Ther.

[24] Kato A, Riesenkampff E, Yim D (2017). Pediatric Fontan patients are at risk for myocardial fibrotic remodeling and dysfunction. Int J Cardiol.

[25] Khoo NS, Smallhorn JF, Kaneko S (2011). Novel insights into RV adaptation and function in hypoplastic left heart syndrome between the first 2 stages of surgical palliation. JACC Cardiovasc Imaging.

[26] Hauser M, Bengel FM, Hager A (2003). Impaired myocardial blood flow and coronary flow reserve of the anatomical right systemic ventricle in patients with congenitally corrected transposition of the great arteries. Heart Br Card Soc.

[27] Zaidi SJ, Penk J, Cui VW, Roberson DA (2019). Right ventricular mechanical dyssynchrony in hypoplastic left heart syndrome: correlation with systolic function and QRS duration. Pediatr Cardiol.

[28] Friedberg MK, Silverman NH, Dubin AM, Rosenthal DN (2007). Right ventricular mechanical dyssynchrony in children with hypoplastic left heart syndrome. J Am Soc Echocardiogr.

[29] Ghelani SJ, Colan SD, Azcue N (2018). Impact of ventricular morphology on fiber stress and strain in Fontan patients. Circ Cardiovasc Imaging.

[30] Hornik CP, He X, Jacobs JP (2011). Complications after the Norwood operation: an analysis of The Society of Thoracic Surgeons Congenital Heart Surgery Database. Ann Thorac Surg.

[31] Cnota JF, Allen KR, Colan S (2013). Superior cavopulmonary anastomosis timing and outcomes in infants with single ventricle. J Thorac Cardiovasc Surg.

[32] Gaynor JW, Mahle WT, Cohen MI (2002). Risk factors for mortality after the Norwood procedure. Eur J Cardio-Thorac Surg.

[33] Aiyagari R, Rhodes JF, Shrader P (2014). Impact of pre-stage II hemodynamics and pulmonary artery anatomy on 12-month outcomes in the Pediatric Heart Network Single Ventricle Reconstruction trial. J Thorac Cardiovasc Surg.

[34] Wilson TG, Shi WY, Iyengar AJ (2017). Twenty-five year outcomes of the lateral tunnel fontan procedure. Semin Thorac Cardiovasc Surg.

[35] Frommelt PC, Hu C, Trachtenberg F (2019). Impact of initial shunt type on echocardiographic indices in children after single right ventricle palliations. Circ Cardiovasc Imaging.

[36] Altmann K, Printz BF, Solowiejczky DE (2000). Two-dimensional echocardiographic assessment of right ventricular function as a predictor of outcome in hypoplastic left heart syndrome. Am J Cardiol.

[37] Colquitt JL, Loar RW, Morris SA (2019). Serial strain analysis identifies hypoplastic left heart syndrome infants at risk for cardiac morbidity and mortality: a pilot study. J Am Soc Echocardiogr Off Publ Am Soc Echocardiogr.

[38] Penk JS, Zaidi SJH, Lefaiver CA (2018). Tissue motion annular displacement predicts mortality/transplant after the bidirectional Glenn. World J Pediatr Congenit Heart Surg.

[39] Alsoufi B, Gillespie S, Kim D (2016). The impact of dominant ventricle morphology on palliation outcomes of single ventricle anomalies. Ann Thorac Surg.

[40] Iyengar AJ, Winlaw DS, Galati JC (2014). The extracardiac conduit Fontan procedure in Australia and New Zealand: hypoplastic left heart syndrome predicts worse early and late outcomes. Eur J Cardio-Thorac Surg.

[41] Josephson CB, Howlett JG, Jackson SD (2006). A case series of systemic right ventricular dysfunction post atrial switch for simple D-transposition of the great arteries: the impact of beta-blockade. Can J Cardiol.

[42] Dore A, Houde C, Chan K-L (2005). Angiotensin receptor blockade and exercise capacity in adults with systemic right ventricles: a multicenter, randomized, placebo-controlled clinical trial. Circulation.

[43] Sachdeva R, Valente AM, Writing Group (2020). J Am Coll Cardiol.

